# The conserved C-terminal residues of FAM83H are required for the recruitment of casein kinase 1 to the keratin cytoskeleton

**DOI:** 10.1038/s41598-022-16153-y

**Published:** 2022-07-12

**Authors:** Takahisa Kuga, Naoki Inoue, Kensuke Sometani, Shino Murataka, Minami Saraya, Rina Sugita, Toshinari Mikami, Yasunori Takeda, Masanari Taniguchi, Kentaro Nishida, Nobuyuki Yamagishi

**Affiliations:** 1 Laboratory of Analytics for Biomolecules, Faculty of Pharmaceutical Science, Setsunan University, 45-1 Nagaotoge-cho, Hirakata-shi, Osaka 573-0101 Japan; 2 Pax Creation Medical Lab., LLC, Iwate, 020-0805 Japan; 3 Division of Clinical Pathology, Department of Reconstructive Oral and Maxillofacial Surgery, School of Dentistry, Iwate Medical University, Iwate, 028-3694 Japan; 4 Department of Integrative Pharmaceutical Sciences, Faculty of Pharmaceutical Science, Setsunan University, Osaka, 573-0101 Japan

**Keywords:** Cell biology, Cell signalling, Cytoskeleton

## Abstract

The casein kinase 1 (CK1) family of serine/threonine protein kinases is involved in diverse cellular events at discrete subcellular compartments. FAM83H acts as a scaffold protein that recruits CK1 to the keratin cytoskeleton or to the nuclear speckles, which are storage sites for splicing factors. We determined the amino acid region of FAM83H required for recruiting CK1 to the keratin cytoskeleton. The subcellular localization of mutant FAM83H proteins with deletions of amino acid residues at different positions was evaluated via immunofluorescence. FAM83H mutants with deleted C-terminal residues 1134–1139, which are conserved among vertebrates, lost the ability to localize and recruit CK1 to the keratin cytoskeleton, suggesting that these residues are required for recruiting CK1 to the keratin cytoskeleton. The deletion of these residues (1134–1139) translocated FAM83H and CK1 to the nuclear speckles. Amino acid residues 1 to 603 of FAM83H were determined to contain the region responsible for the recruitment of CK1 to the nuclear speckles. Our results indicated that FAM83H recruits CK1 preferentially to the keratin cytoskeleton and alternatively to the nuclear speckles.

## Introduction

The casein kinase 1 (CK1) family are serine/threonine protein kinases^1–3^. Seven CK1 isoforms have been identified in humans, namely, α, α-like, δ, ε, γ1, γ2, and γ3. CK1 is involved in diverse cellular processes, including circadian rhythm, Wnt signaling, membrane trafficking, cytoskeleton maintenance, DNA replication, DNA damage response, RNA metabolism, and parasitic infections^1–4^. As expected from its diverse functions, various CK1 substrates have been reported^1^. These substrates are distributed in diverse subcellular compartments; thus, CK1 needs to localize in the proper subcellular compartments to phosphorylate their corresponding substrates. CK1 has been reported to localize in diverse subcellular compartments, including the cytoplasm, centrosomes, microtubules, nucleus, membrane structures, mitochondria, and keratin cytoskeleton^1–7^. Some proteins have been identified as scaffolds that recruit or sequestrate CK1 to specific subcellular compartments^1^.

FAM83H is one of the scaffold proteins that dictate CK1 subcellular localization. FAM83H recruits CK1 to the keratin cytoskeleton or to nuclear speckles, which are storage sites for splicing factors^5–7^. FAM83H was originally identified as a protein responsible for the formation of dental enamel because autosomal-dominant hypocalcified amelogenesis imperfecta (ADHCAI) is caused by a mutation in the FAM83H gene^8^. In addition, a variety of reports have suggested the involvement of FAM83H in cancer^5,9–14^.

FAM83H regulates the organization of the keratin cytoskeleton by recruiting CK1 ^5,7,15–17^. FAM83H overexpression changes the structure of the keratin cytoskeleton from filamentous to speckle-like structures, possibly through the excessive recruitment of CK1 to the keratin cytoskeleton^5^. On the other hand, knockdown of FAM83H thickens the bundle of keratin filaments, possibly through the recruitment of less CK1 to the keratin cytoskeleton^5^.

In nuclear speckles, FAM83H and CK1 may be involved in processing mRNAs because their localization in nuclear speckles depends on SON, an mRNA splicing cofactor present in nuclear speckles^6^. CK1α is associated with and phosphorylates some splicing factors known as SR proteins and a nuclear poly(A) polymerase, Star-PAP, in nuclear speckles^18,19^. It has been shown that CK1α is required for Star-PAP target mRNA synthesis^19^.

FAM83H concomitantly binds to CK1 and keratins or to SON to recruit CK1 to the keratin cytoskeleton or to the nuclear speckles, respectively. CK1, keratins, and SON are detected in FAM83H-FLAG immunoprecipitates^5,6^. The N-terminal DUF1669 domain of FAM83H is responsible for binding to CK1^5,20,21^. Amino acid residues 1–286 of FAM83H are sufficient to bind to CK1^5^. Amino acid residues 287–1179 of FAM83H contain the region responsible for binding to keratins and SON^5,6^; however, detailed positions of the region have not yet been identified. It is also unknown whether the amino acid position of FAM83H responsible for binding to keratins is the same or different for binding to SON.

In ADHCAI patients, truncated FAM83H proteins are translated from mutant *FAM83H*, which contain a premature termination codon^8,22^. All the truncations reported occur between amino acid residues 287 and 694^23^. An important question is whether these truncated proteins contain the residues responsible for binding to keratins or SON; that is, whether they can recruit CK1 to the keratin cytoskeleton or to the nuclear speckles. In the present study, we searched for the amino acid region of FAM83H required for recruiting CK1 to the keratin cytoskeleton.

## Results

### The C-terminal half of FAM83H is required for its localization in the keratin cytoskeleton

**Figure 1:**
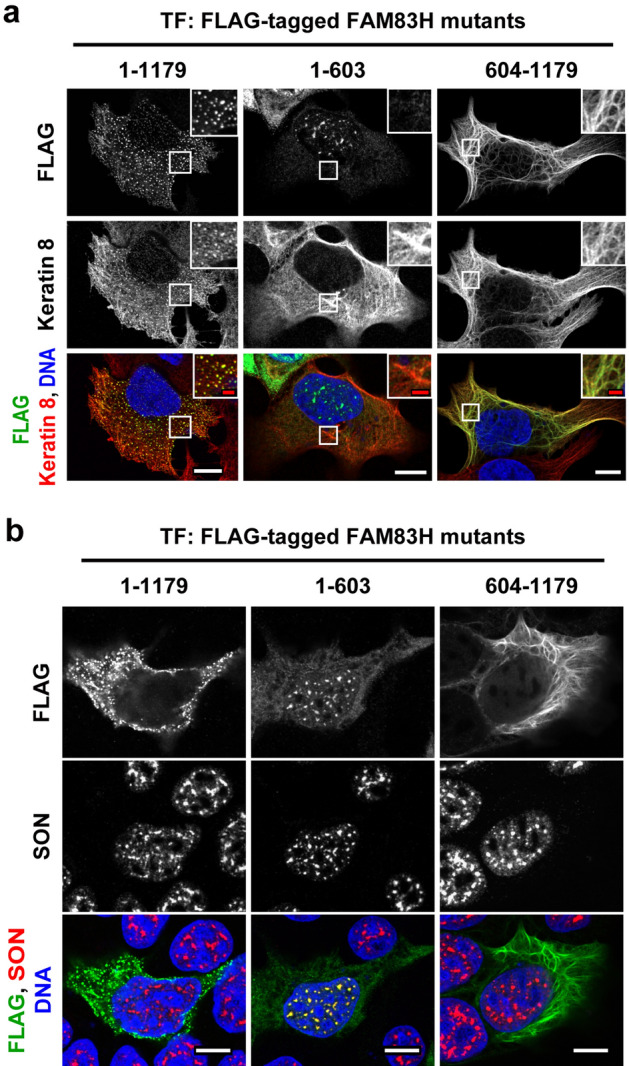
The C-terminal half of FAM83H is required for its localization in the keratin cytoskeleton. DLD1 cells were transfected with the plasmids encoding the indicated FAM83H mutants and were analyzed via immunofluorescence using antibodies for (**a**) FLAG (green) and keratin 8 (red) or (**b**) for FLAG (green) and SON (red). Nuclei were visualized by DAPI (blue). (**a**) Magnified images at the regions enclosed by the white squares are shown. White and red scale bars indicate 10 and 2 μm, respectively.

To determine which of the N- or C-terminal half of FAM83H is required for its localization in the keratin cytoskeleton, we tested the subcellular localization of the following fragment proteins of FAM83H: an N-terminal half (amino acid residues 1–603) and a C-terminal half (amino acid residues 604–1179). These fragment proteins are C-terminally tagged with the FLAG epitope. DLD1 colorectal cancer cells were transfected with plasmids encoding these fragment proteins or the full length of FAM83H (amino acid residues 1–1179). Then, their subcellular localization was evaluated via immunofluorescence with anti-FLAG and anti-keratin 8 antibodies (Fig. 1a). As previously demonstrated^5^, overexpression of the full length of FAM83H partially changed the structure of the keratin cytoskeleton from filamentous to speckle-like structures (Fig. 1a). The full length of FAM83H showed co-localization with the speckle-like structures of keratins (Fig. 1a). Overexpression of the N- or C-terminal half of FAM83H did not have an obvious impact on the morphology of the keratin cytoskeleton (Fig. 1a), indicating that the halves alone are not sufficient to regulate the morphology of the keratin cytoskeleton. The C-terminal half of FAM83H localized in the keratin cytoskeleton (Fig. 1a). The N-terminal half of FAM83H did not co-localize with the keratin cytoskeleton but rather localized in nuclear speckles, which was evaluated via co-localization with SON (Fig. 1a,b). These results indicate that the C-terminal half of FAM83H contains an amino acid region required for its localization in the keratin cytoskeleton.

### Amino acid residues 1135–1139 of FAM83H are required for its localization in the keratin cytoskeleton

**Figure 2:**
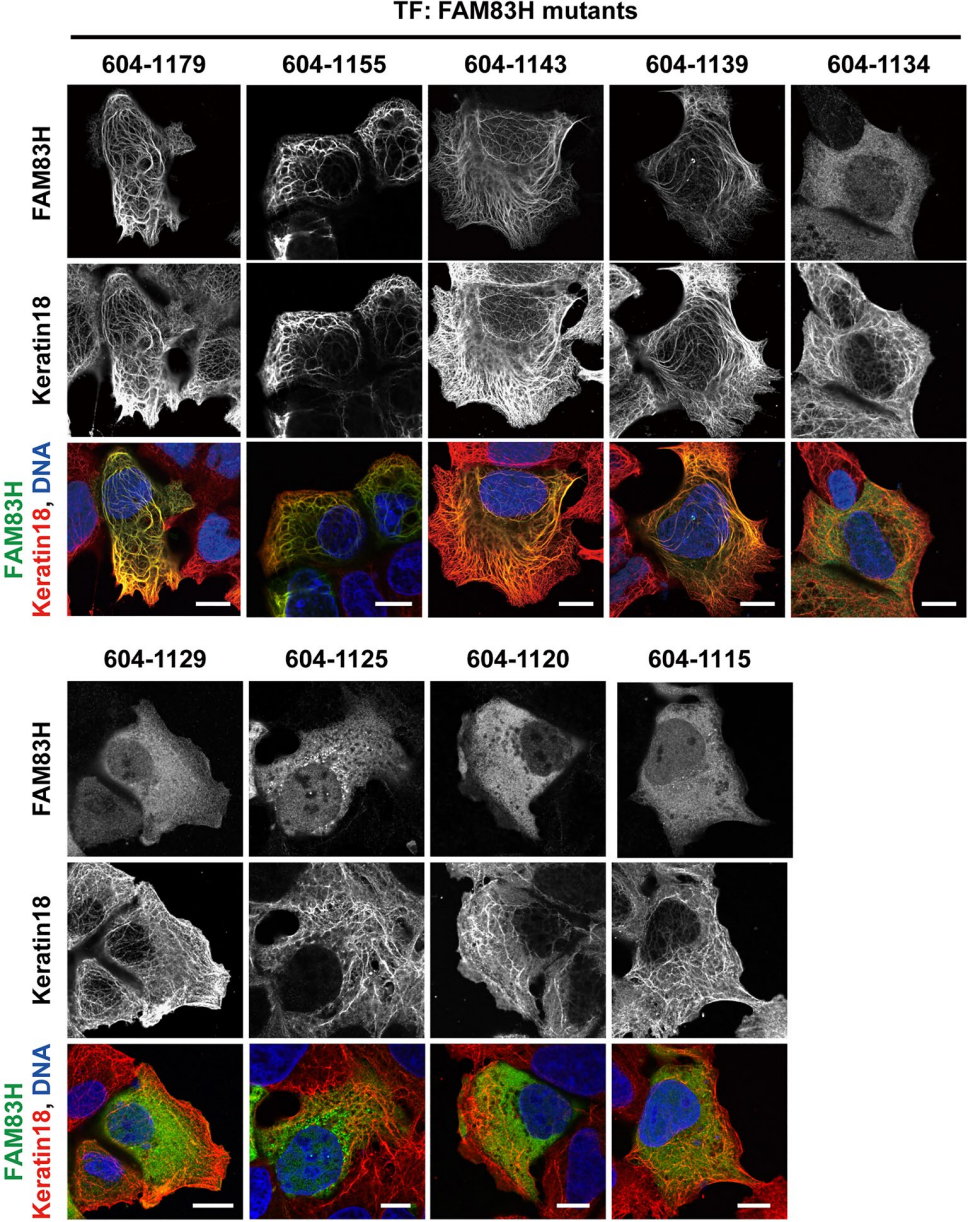
Amino acid residues 1134–139 of FAM83H are required for its localization in the keratin cytoskeleton. DLD1 cells were transfected with the plasmids encoding the indicated FAM83H mutants and were analyzed via immunofluorescence using antibodies for FAM83H (green) and keratin 18 (red). Nuclei were visualized by DAPI (blue). Bars, 10 μm.

The amino acid region of FAM83H required for its localization in the keratin cytoskeleton was further narrowed down by testing subcellular localization of the C-terminal halves of FAM83H with different lengths of deletion at the C-terminal end (Fig. 2). Four mutants, namely, 604–1179, 604–1155, 604–1143, and 604–1139, co-localized with the keratin cytoskeleton. In sharp contrast, five mutants, namely, 604–1134, 604–1129, 604–1125, 604–1120, and 604–1115, did not co-localize with the keratin cytoskeleton. These results indicate that amino acid residues 1135–1139 of FAM83H are required for its localization in the keratin cytoskeleton. These five residues, 1135–1139, are thereafter called the keratin-localization (KL) residues.

**Figure 3:**
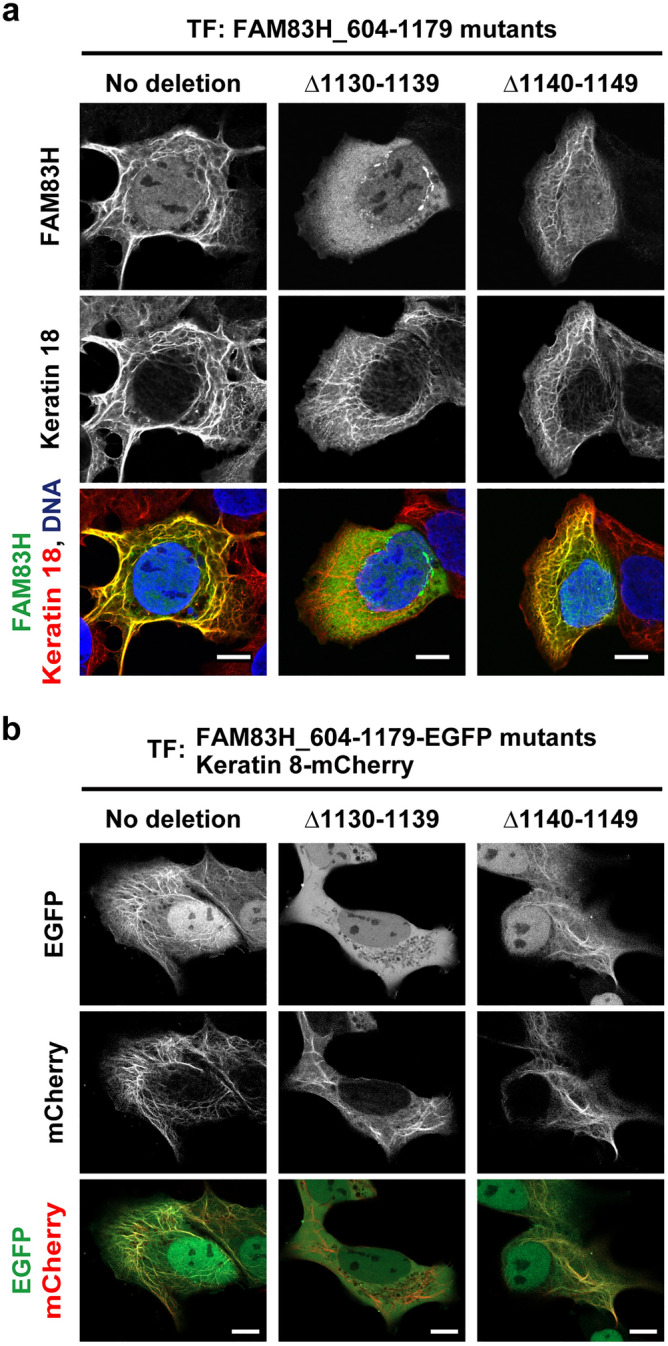
Deletion of 10 amino acid residues 1130–1139 of FAM83H results in its delocalization from the keratin cytoskeleton. (**a**) DLD1 cells were transfected with the plasmids encoding the indicated FAM83H mutants and were analyzed via immunofluorescence using antibodies for FAM83H (green) and keratin 18 (red). Nuclei were visualized by DAPI (blue). (**b**) DLD1 cells were co-transfected with plasmids encoding keratin 8-mCherry (red) and the indicated EGFP-fused FAM83H mutants (green) and analyzed via live cell imaging. Bars, 10 μm.

We next compared the localization of the FAM83H mutant 604–1179 with ten deleted amino acid residues between 1130 and 1139 (Δ1130–1139) or between 1140 and 1149 (Δ1140–1149). FAM83H_604-1179Δ1130–1139 does not have the KL residues. As expected, 604–1179Δ1130–1139 was unable to localize in the keratin cytoskeleton, whereas 604–1179Δ1140–1149 was still able to (Fig. 3a). Furthermore, we performed live cell imaging of cells co-transfected with plasmids encoding C-terminally EGFP-tagged FAM83H mutants and C-terminally mCherry-tagged keratin 8. FAM83H_604-1179-EGFP and 604–1179Δ1140–1149-EGFP co-localized with keratin 8-mCherry, whereas 604–1179Δ1130–1139-EGFP did not (Fig. 3b). These results support the requirement of the KL residues for FAM83H to localize in the keratin cytoskeleton. Intriguingly, the KL residues are within the amino acid region 1123–1141 conserved in vertebrates^24^.

### Deletion of the KL residues results in the translocation of FAM83H and CK1α from the keratin cytoskeleton to the nuclear speckles

**Figure 4:**
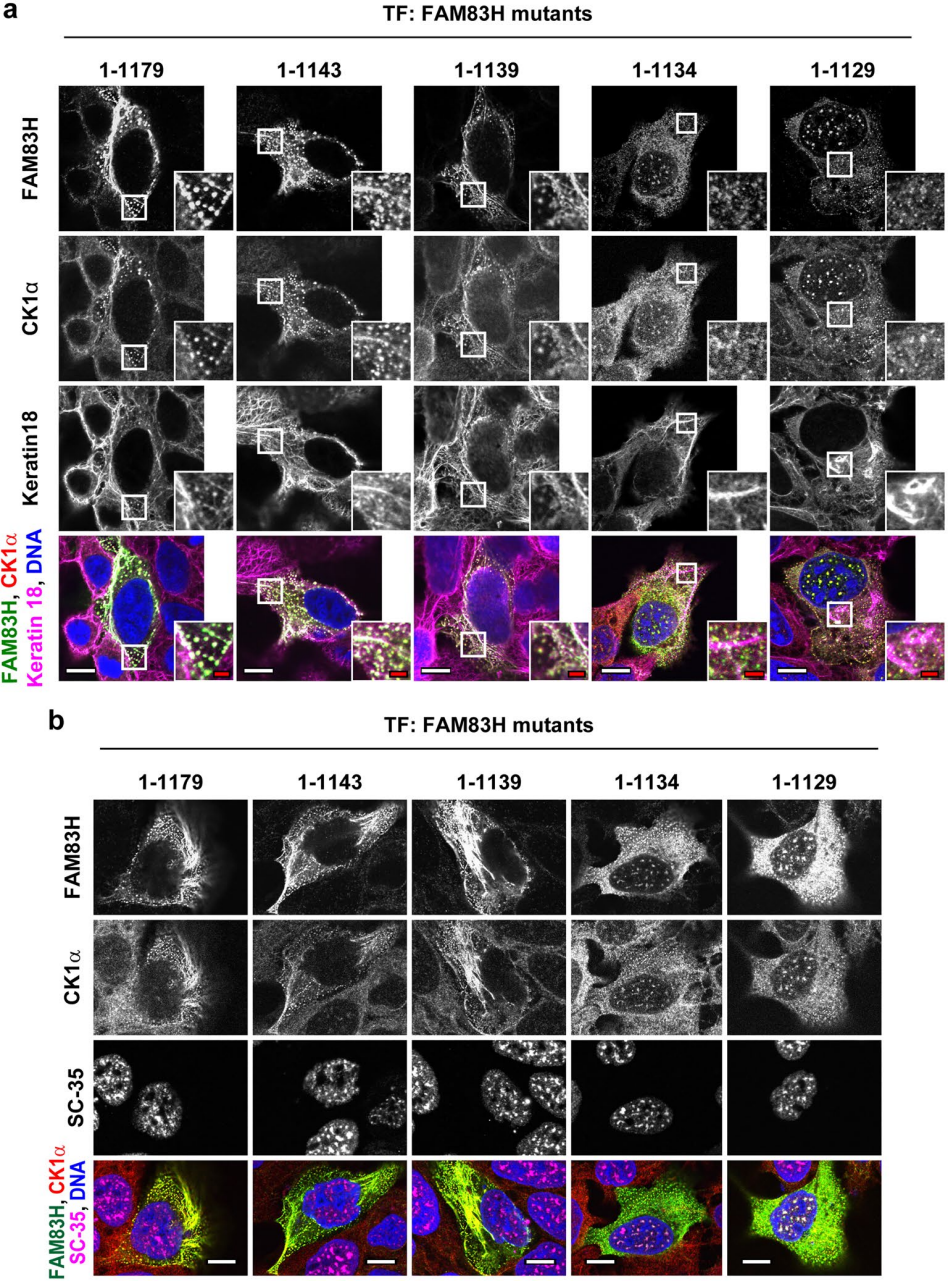
Deletion of amino acid residues 1134–1139 of FAM83H results in its translocation from the keratin cytoskeleton to the nuclear speckles. DLD1 cells were transfected with the plasmids encoding the indicated FAM83H mutants and were analyzed via immunofluorescence using antibodies for (**a**) FAM83H (green), CK1α (red), and keratin 18 (magenta) or (**b**) FAM83H (green), CK1α (red), and SC-35 (magenta). Nuclei were visualized by DAPI (blue). (**a**) Magnified images at the regions enclosed by white squares are shown. White and red scale bars indicate 10 and 2 μm, respectively.

Our previous study suggested that FAM83H more commonly localizes in the keratin cytoskeleton than in the nuclear speckles^6^. To substantiate this concept, we tested whether the deletion of the KL residues triggers the translocation of FAM83H and CK1 from the keratin cytoskeleton to the nuclear speckles. We generated plasmids encoding mutants of FAM83H: 1–1143, 1–1139, 1–1134, and 1–1129. These mutants possess the N-terminal half (amino acid residues 1–603), which has been shown to be responsible for localization in nuclear speckles (Fig. 1b). The mutants possessing the KL residues, mutants 1–1143 and 1–1139, and the wild-type 1–1179 localized in the keratin cytoskeleton but not in the nuclear speckles (Fig. 4a,b). Conversely, the mutants without the KL residues, mutant 1–1134 and 1–1129, localized in the nuclear speckles but not in the keratin cytoskeleton (Fig. 4a,b). In mutants 1–1143 and 1–1139 and the wild-type, CK1α also localized in the keratin cytoskeleton. In contrast, in mutants 1–1134 and 1–1129, CK1α localized in the nuclear speckles (Fig. 4a,b).

**Figure 5:**
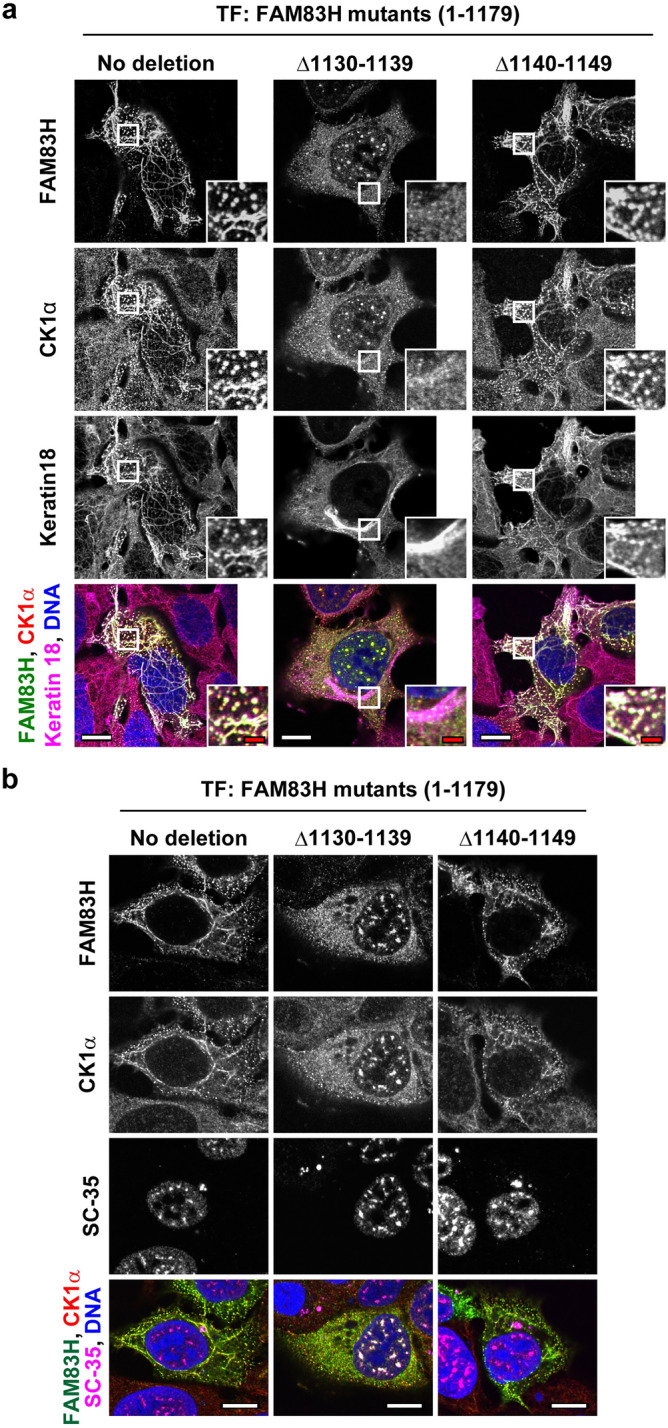
Deletion of 10 amino acid residues 1130–1139 of FAM83H is sufficient for its translocation from the keratin cytoskeleton to the nuclear speckles. DLD1 cells were transfected with the plasmids encoding the indicated FAM83H mutants and were analyzed via immunofluorescence using antibodies for (**a**) FAM83H (green), CK1α (red), and keratin 18 (magenta) or (**b**) FAM83H (green), CK1α (red), and SC-35 (magenta). Nuclei were visualized by DAPI (blue). (**a**) Magnified images at the regions enclosed by white squares are shown. White and red bars indicate 10 and 2 μm, respectively.

We next tested the subcellular localization of the full length of FAM83H with 10 deleted amino acid residues from 1130 to 1139 (Δ1130–1139) and from 1140 to 1149 (Δ1140–1149). FAM83HΔ1130–1139 lacks the KL residues, whereas FAM83HΔ1140–1149 retains them. FAM83HΔ1140–1149 co-localized with the speckle-like structures of keratins and recruited CK1α to the keratin structures, whereas FAM83HΔ1130–1139 localized in the nuclear speckles and recruited CK1α (Fig. 5a,b). Taken together, our results indicate that the deletion of the KL residues results in the translocation of FAM83H and CK1α from the keratin cytoskeleton to the nuclear speckles.

**Figure 6:**
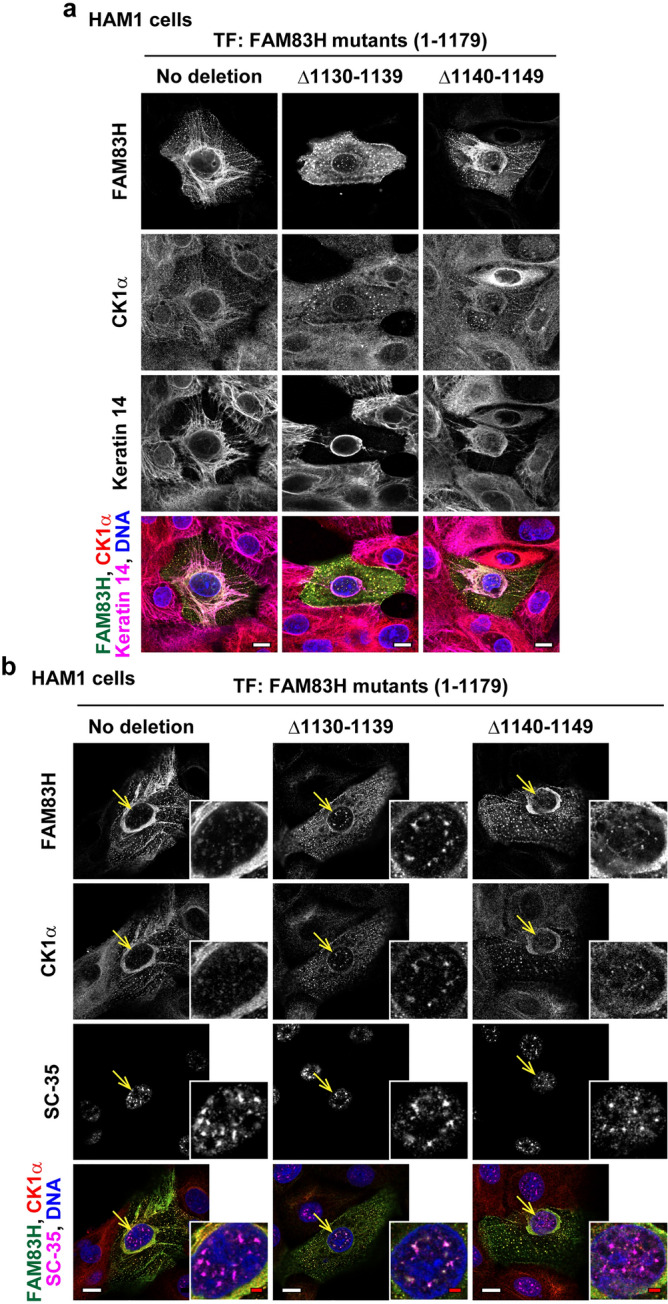
Immunofluorescence of human ameloblastoma HAM1 cells. HAM1 cells were transfected with the plasmids encoding the indicated FAM83H mutants and were analyzed via immunofluorescence using antibodies for (**a**) FAM83H (green), CK1α (red), and keratin 14 (magenta) or (**b**) FAM83H (green), CK1α (red), and SC-35 (magenta). Nuclei were visualized by DAPI (blue). (**b**) Magnified images of the nuclei pointed by yellow arrows are shown. White and red bars indicate 10 and 2 μm, respectively.

Furthermore, we determined the subcellular localization of FAM83HΔ1130–1139 and Δ1140–1149 in human ameloblastoma HAM1 cells (Fig. 6), and normal epidermal keratinocyte PSVK1 cells (Fig. S1). DLD1 cells expressed simple epithelial keratins, i.e., type II keratin 8 and type I keratin 18 (Fig. S2). HAM1 and PSVK1 cells expressed type II keratin 5 and type I keratin 14, although keratins 8 and 18 are also expressed in these cells (Fig. S2). Keratins 5 and 14 are known to be expressed in normal ameloblasts^25^ and epidermal basal cells^16,17^. Aberrant morphology of the keratin cytoskeleton was observed in HAM1 and PSVK1 transfectants, irrespective of the kind of transfected mutants (Figs. 6a and S1a). In HAM1 and PSVK1 cells, wild-type FAM83H and FAM83HΔ1140–1149, but not FAM83HΔ1130–1139, localized and recruited CK1α to the keratin cytoskeleton (Figs. 6a and S1a). In addition, the translocation of FAM83HΔ1130–1139 into the nuclear speckles was observed in HAM1 cells to a milder level than in DLD1 cells (Figs. 5b and 6b), whereas it was little observed in PSVK1 cells (Fig. S1b). Overall, these results indicate that the recruitment of CK1α to the keratin cytoskeleton is commonly regulated by FAM83H in multiple cell types. Moreover, the recruitment of CK1α to the nuclear speckles is regulated by FAM83H in a cell type-dependent manner.

## Discussion

**Figure 7:**
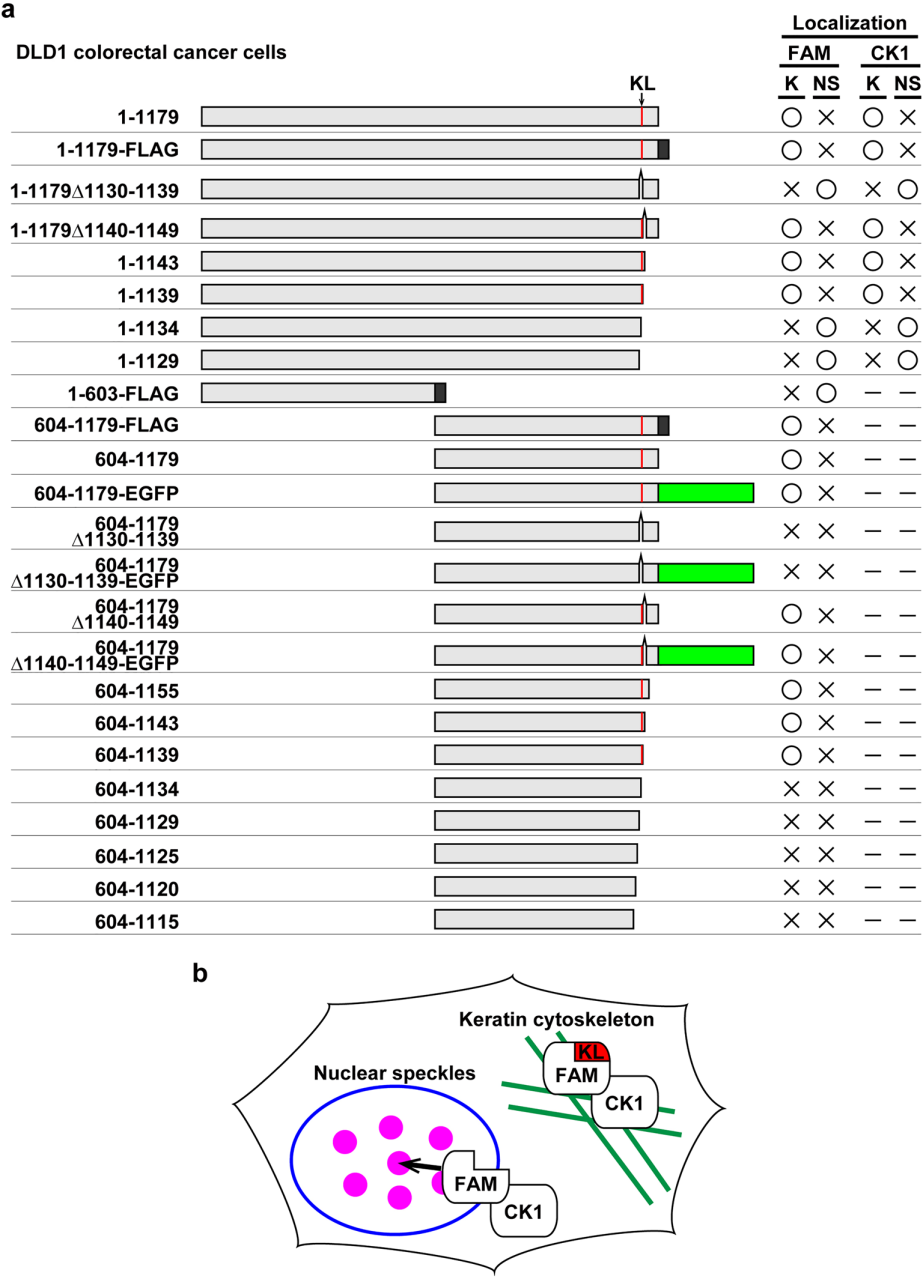
The FAM83H mutants used in the study. (**a**) The full length of FAM83H is composed of 1179 amino acid residues. Light gray columns indicate the amino acid residues of FAM83H. Dark gray columns indicate the amino acid residues of the FLAG-tag. Green columns indicate the amino acid residues of EGFP. Red lines indicate the position of the keratin-localization (KL) residues (1134–1139). The open circle and  cross  marks indicate the positive and negative localizations of FAM83H and the recruitment of CK1α to the keratin cytoskeleton (K) or nuclear speckles (NS) in DLD1 cells, respectively. The dash mark indicates that was not tested. (**b**) Schema. FAM83H mutants with the KL residues localize and recruit CK1 to the keratin cytoskeleton, whereas mutants without the KL residues localize and recruit CK1 to the nuclear speckles.

In the present study, we demonstrated that the C-terminal amino acid residues 1134–1139 of FAM83H, termed here the KL residues, are required for its localization and for the recruitment of CK1 to the keratin cytoskeleton (Fig. 7). We further revealed that the deletion of the KL residues results in the translocation of FAM83H and CK1 from the keratin cytoskeleton to the nuclear speckles. These findings are important for understanding the mechanism of ADHCAI because truncated proteins translated from the mutant *FAM83H* lack the KL residues. This study hypothesized that the delocalization of FAM83H and CK1 from the keratin cytoskeleton may trigger cellular events that prevent dental enamel calcification.

The KL residues may be part of the functional unit for localization in the keratin cytoskeleton. Evolutionary analysis of FAM83H determined that amino acid residues 1123–1141 (M^1123^ESMRKEKRVYSRFEVFCK^1141^) are highly conserved among vertebrates^24^. The KL residues (R^1135^FEVF^1139^) are positioned at the N-terminal end of this conserved region. The conserved amino acid residues 1123–1141 may constitute the fully functional unit for localization in the keratin cytoskeleton.

It is possible to speculate that the KL residues are involved in the binding of FAM83H to keratin proteins. For instance, FAM83H has been demonstrated to bind concomitantly to both CK1 and keratins linking these proteins and resulting in the recruitment of CK1 to the keratin cytoskeleton^5^. Experiments using co-immunoprecipitation revealed that amino acid residues 1–286 of FAM83H are responsible for binding to CK1, whereas amino acid residues 287–1179 are responsible for binding to keratins^5^. We performed co-immunoprecipitation experiments of FLAG-tagged FAM83H_604-1179 without the KL residues, Δ1130–1139; however, keratins were co-precipitated with it (Fig. S3). Inferentially, this preliminary result suggests that the KL residues are not involved in the binding of FAM83H to keratin proteins. Therefore, there is a need for further experiments to validate these preliminary results.

The present study demonstrated that distinct amino acid regions of FAM83H are responsible for its localization in the keratin cytoskeleton or in nuclear speckles. Previous studies have shown that amino acid residues 287–1179 of FAM83H contain the regions responsible for its localization in these subcellular compartments^5,6^; however, the specific positions of these regions remain unknown. As described above, the KL residues are responsible for localization in the keratin cytoskeleton. On the other hand, the N-terminal half of FAM83H (amino acid residues 1–603) was demonstrated to contain the region responsible for localization in nuclear speckles (Fig. 1). A previous study has demonstrated that the localization of FAM83H in nuclear speckles depends on its binding to SON^6^. These results suggest that the N-terminal half (amino acid residues 1–603) of FAM83H contains the region responsible for binding to SON.

This study supports the concept that FAM83H preferentially localizes in the keratin cytoskeleton than in nuclear speckles. A previous study showed that transfection with siRNA for keratins 18 and 19 resulted in the translocation of FAM83H and CK1α from the keratin cytoskeleton to the nuclear speckles^6^. Furthermore, the results in this study indicated that the deletion of the KL residues induced their translocation from the keratin cytoskeleton to the nuclear speckles (Figs. 4 and 5). These results suggest that FAM83H localizes in the nuclear speckles and recruits CK1 only when FAM83H cannot localize in the keratin cytoskeleton.

It is possible that in ADHCAI patients, FAM83H mutants cannot localize in the keratin cytoskeleton. Mutant *FAM83Hs* in ADHCAI patients encode for mutant proteins prematurely truncated between amino acid residues 287 and 694^23^. This means that all the truncated proteins lack the KL residues (1134–1139) and are possibly unable to localize in the keratin cytoskeleton. However, this may not always mean that the truncated proteins translocate to the nuclear speckles. Although the truncated protein with amino acid residues 1–603 of FAM83H was shown to localize in the nuclear speckles (Fig. 1), the truncated protein with amino acid residues 1–286, the shortest mutant among those reported, did not localize in the nuclear speckles^6^. The delocalization, but not the translocation, of FAM83H and CK1 to the nuclear speckles may be required for the development of ADHCAI.

Epithelial to mesenchymal transition (EMT) of cancer can also induce the translocation of FAM83H and CK1 from the keratin cytoskeleton to the nuclear speckles. EMT is involved in cancer metastasis. One characteristic of EMT is a decreased expression of keratins^26^. In human colorectal cancer tissues, poor organization of the keratin cytoskeleton was observed in the invasive front, where FAM83H localized in nuclear speckles^6^. Some colorectal cancer cell lines lacking epithelial keratin expression, such as RKO and WiDr cells, showed the localization of FAM83H and CK1 in nuclear speckles^6^. These results indicate that the decreased expression of epithelial keratins during EMT leads to the translocation of FAM83H and CK1 to nuclear speckles. CK1α is known to be associated with and to phosphorylate some proteins related to splicing or mRNA synthesis^18,19^. In future studies, the influence of the translocation of FAM83H and CK1 to nuclear speckles on the processing of mRNA for proteins related to cancer metastasis and amelogenesis can be examined.

## Methods

### Plasmids

The p3xFLAG-CMV14-FAM83H-FLAG vector has been previously generated^5^. To generate the p3xFLAG-CMV14-FAM83H (without the FLAG-tag) vector, (1) a PCR fragment was amplified using the forward primer, CATCACCGTTGCCAGCCACAG, the reverse primer, CCGGGATCCTCACTTCTTGCTTTTGAACG, and the p3xFLAG-CMV14-FAM83H-FLAG vector as the template, (2) the PCR fragment was digested by XhoI and BamHI, and (3) the digested fragment was ligated into the p3xFLAG-CMV14-FAM83H-FLAG vector digested by XhoI and BamHI. To generate the p3xFLAG-CMV14-FAM83H_604-1179-FLAG vector, (1) a PCR fragment was amplified using the forward primer, ATAGAATTCACCATGGAAGCGGAGGCTTAC, the reverse primer, TCCAGCAGGCAGCTCTCGAGGCTG, and the p3xFLAG-CMV14-FAM83H-FLAG vector as the template, (2) the PCR fragment was digested by EcoRI and XhoI, and (3) the digested fragment was ligated into the p3xFLAG-CMV14-FAM83H-FLAG vector digested by EcoRI and XhoI. To generate p3xFLAG-CMV14-FAM83H_604-1179 (without the FLAG-tag) vector, the appropriate codon of the p3xFLAG-CMV14-FAM83H_604-1179-FLAG vector was changed into a STOP codon by site-directed mutagenesis using the primers AAGAAGTGAGGATCCCGGGCTGACTAC and GGATCCTCACTTCTTGCTTTTGAAC (according to the manual of the PrimeSTAR Mutagenesis Basal Kit; Takara Bio Inc., Shiga, Japan). To generate p3xFLAG-CMV14-FAM83H_1-603-FLAG, (1) a PCR fragment was amplified using the forward primer, TTGCGGCCGCGAATTCAACA, the reverse primer, AGTCAGCCCGGGATCCGGGCGCCGGTAGGCCGTCGTCGCCCCCATC, and the p3xFLAG-CMV14-FAM83H-FLAG vector as the template, and (2) the PCR fragment was inserted into the p3xFLAG-CMV14 vector (Merck, Darmstadt, Germany) digested by EcoRI and BamHI, according to the manual of the InFusion HD Cloning Kit (Takara Bio Inc.). To generate the p3xFLAG-CMV14-FAM83H_1-1129, 1–1134, 1–1139, 1–1143, 604–1120, 604–1125, 604–1129, 604–1134, 604–1139, and 604–1143 (without the FLAG-tag) vectors, (1) PCR fragments were amplified using the forward primer, GCGCCGCAGCCTCGAGAGCT, the reverse primers listed below, and the p3xFLAG-CMV14-FAM83H_604-1179 vector as the template, and (2) the PRC fragments were inserted using an InFusion HD Cloning Kit into the p3xFLAG-CMV14-FAM83H-FLAG or 604–1179-FLAG vectors digested by XhoI and BamHI. The reverse primers used were AGTCAGCCCGGGATCCTACAGCAGCCGATCGCGCTCCTCCGCGCTGGC for 604–1120, AGTCAGCCCGGGATCCTAGCTCTCCATGCGGCGCAGCAGCCGATCGCG for 604–1125, AGTCAGCCCGGGATCCTACTCCTTGCGCATGCTCTCCATGCGGCGCAG for 1–1129 and 604–1129, AGTCAGCCCGGGATCCTAGCTGTACACGCGCTTCTCCTTGCGCATGCT for 1–1134 or 604–1134, AGTCAGCCCGGGATCCTAGAAGACCTCGAAGCGGCTGTACACGCGCTT for 1–1139 and 604–1139, AGTCAGCCCGGGATCCTACTCTTTCTTGCAGAAGACCTCGAAGCGGCT for 1–1143 and 604–1143, and AGTCAGCCCGGGATCCTACGCGGGGCCTTCCCCTGCCCCAGGGCTGCT for 604–1155. The p3xFLAG-CMV14-FAM83H_604-1115 and 604–1155 (without the FLAG-tag) vectors were generated by replacing the appropriate codons of the p3xFLAG-CMV14-FAM83H_604-1179-FLAG vector with a STOP codon via site-directed mutagenesis using the primers GAGGAGTGAGATCGGCTGCTGCGCCGC and CCGATCTCACTCCTCCGCGCTGGCTGG for 604–1115 and CCCGCGTAGGAGGGCACCAGGGACAGC and GCCCTCCTACGCGGGGCCTTCCCCTGC for 604–1155. The p3xFLAG-CMV14-FAM83H_604-1179Δ1130–1139 and 604–1179Δ1140–1149 (without the FLAG-tag) vectors were generated by deleting the appropriate regions from the p3xFLAG-CMV14-FAM83H_604-1179 (without the FLAG-tag) vector via site-directed mutagenesis using the primers CAAGGAGTGCAAGAAAGAGGAGGCC and TTCTTGCACTCCTTGCGCATGCTCTC for Δ1130–1139 and GGTCTTCGCAGGGGAAGGCCCCGCG and TCCCCTGCGAAGACCTCGAAGCGGCT for Δ1140–1149. To generate the p3xFLAG-CMV14-FAM83H_604-1179Δ1130–1139-FLAG and 604–1179Δ1140–1149-FLAG vectors, (1) PCR fragments were amplified using the primers GCGCCGCAGCCTCGAGAGCT and AGTCAGCCCGGGATCCTCCCTTCTTGCTTTTGAACGTGCC and the p3xFLAG-CMV14-FAM83H_604-1179Δ1130–1139 or 604–1179Δ1140–1149 (without the FLAG-tag) vector as the template, and (2) the PCR fragments were inserted using an InFusion HD Cloning Kit into the p3xFLAG-CMV14-FAM83H_604-1179-FLAG digested by XhoI and BamHI. To generate p3xFLAG-CMV14-FAM83H_1-1179Δ1130–1139 and 1–1179Δ1140–1149 (without the FLAG-tag) vectors, (1) PCR fragments were amplified using the primers GCGCCGCAGCCTCGAGAGCT and AGTCAGCCCGGGATCCTCAC and the p3xFLAG-CMV14-FAM83H_604-1179Δ1130–1139 or 604–1179Δ1140–1149 (without the FLAG-tag) vector as the template, and (2) the PCR fragments were inserted using an InFusion HD Cloning Kit into the p3xFLAG-CMV14-FAM83H (without the FLAG-tag) digested by XhoI and BamHI. To generate the pEGFP-N1-FAM83H_604-1179, 604–1179Δ1130–1139, and 604–1179Δ1140–1149 vectors, (1) PCR fragments were amplified using the primers TCTCGAGCTCAAGCTTAACATGGAAGCGGAGGCTTATGAAGACGAC and GGCGACCGGTGGATCCCCCTTCTTGCTTTTGAACGTGCCCAGGATC and the p3xFLAG-CMV14-FAM83H_604-1179, 604–1179Δ1130–1139 or 604–1179Δ1140–1149 vector (without the FLAG-tag) as the template, and (2) the PCR fragments were inserted using an InFusion HD Cloning Kit into the pEGFP-N1 vector (Takara Bio Inc.) digested by BamHI and HindIII. To generate the pmCherry-N1-keratin 8 vector, (1) PCR fragments were amplified using the primers TCTCGAGCTCAAGCTTACCATGTCCATCAGGGTGACCCAGAAGTC and GGCGACCGGTGGATCCCCCTTGGGCAGGACGTCAGAGGACTCAGACAC and the pOTB7-keratin 8 (Genome Network Progject Clone IRAL005B16; RIKEN BRC, Ibaraki, Japan)^27–30^ as the template, and (2) the PCR fragments were inserted using an InFusion HD Cloning Kit into the pmCherry-N1 vector (Takara Bio Inc.) digested by BamHI and HindIII. Restriction enzymes were purchased from New England Biolabs (MA, USA). Primers were synthesized by Eurofins Genomics Inc. (Tokyo, Japan).

### Cell culture and transfection

DLD1 colorectal cancer cells were obtained from the American Type Culture Collection (VA, USA). DLD1 cells were cultured in Dulbecco’s Modified Eagle Medium (DMEM; Nacalai Tesque, Kyoto, Japan) supplemented with 5% fetal bovine serum (FBS; PAA Laboratories GmbH, Pasching, Austria). HAM1 human ameloblastoma cells were previously established^31^. HAM1 cells were cultured in Keratinocyte-SFM Medium (K-SFM; Thermo Fisher Scientific, MA, USA). PSVK1 human normal foreskin keratinocytes were obtained from the JCRB Cell Bank (JCRB1093; Osaka, Japan)^32^. PSVK1 cells were cultured in Keratinocyte Media 2 (PromoCell, Heidelberg, Germany). Cells were maintained at 37 °C in 5% CO_2_. Transfection with a plasmid was performed using Lipofectamine 2000 (Thermo Fisher Scientific) for DLD1 cells and Lipofectamine 3000 (Thermo Fisher Scientific) for HAM1 and PSVK1 cells. The transfection using Lipofectamine 3000 was performed as described previously^7^.

### Antibodies

The following antibodies were used: anti-FLAG (F1804; Merck), anti-keratin 5 (RM-2106-S0; Thermo Fisher Scientific), anti-keratin 8 (ab53280; Abcam, Cambridge, UK), anti-keratin 14 (MS-115-P0; Thermo Fisher Scientific), anti-keratin 18 (MS-142-P0; Thermo Fisher Scientific), anti-SON (HPA023535; Merck), anti-FAM83H (HPA024604; Merck), anti-CK1α (sc-6477; Santa Cruz Biotechnology, CA, USA), anti-GAPDH (2275-PC-100; R&D Systems, MN, USA), and anti-SC-35 (S4045; Merck). Alexa Fluor 488-conjugated donkey anti-mouse IgG (A-21202) and donkey anti-rabbit IgG (A-21206), Alexa Fluor 568-conjugated donkey anti-mouse IgG (A10037) and donkey anti-rabbit IgG (A10042), Alexa Fluor 594-conjugated donkey anti-goat IgG (A-11058), and Alexa Fluor 647-conjugated donkey anti-mouse IgG (A-31571) were purchased from Thermo Fisher Scientific. Horseradish peroxidase-conjugated anti-mouse IgG (#7076; Cell Signaling Technology, MA, USA) and anti-rabbit IgG (711-035-152; Jackson Immunoresearch, PA, USA) antibodies were used for Western blotting.

### Immunofluorescence and live cell imaging

The cultured cells were fixed with 100% Methanol at − 20 °C for 2 min, blocked with Blocking One (Nacalai Tesque) on ice for 30 min, and sequentially incubated with primary and secondary antibodies at room temperature for 1 h. DNA was stained with 100 ng/mL of 4’-6-diamidino-2-phenylindole (DAPI). The stained samples were viewed under an FV1000 confocal microscope equipped with the objective lens UPlan SApo 100× /1.40 Oil (Olympus, Tokyo, Japan) or an LSM900 Airyscan2 equipped with the objective lens Plan-Apochromat 63x/1.40 Oil DIC (Carl Zeiss AG, Oberkochen, Germany). For live cell imaging, cells cultured on a glass-bottom dish were viewed under an LSM900 Airyscan2. All images shown in figures are single confocal planes of representative cells. In all experiments, at least three images of different optical aeras were taken. Composite images were prepared using Photoshop CS5 (Adobe, CA, USA).

### Immunoprecipitation and Western blotting

For preparation of cell lysates used for immunoprecipitation (IP lysates), cells were suspended in PBS containing 1% NP40 (Nacalai Tesque) and protease inhibitor cocktail (25955-11; Nacalai Tesque), and then homogenized by sonication (Bioruptor; Cosmo Bio Co., Ltd, Tokyo, Japan). After centrifugation at 100,000×*g* for 30 min, the supernatant was collected. Immunoprecipitation was performed using anti-FLAG antibody crosslinked to Protein G Dynabeads (Thermo Fisher Scientific) using dimethyl pimelimidate dihydrochloride (Nacalai Tesque). IP lysates were reacted with antibody-coated Dynabeads for 1 h at 4 °C, and the absorbed proteins were eluted with 100 mM glycine–HCl (pH 2.8).

For the extraction of whole cellular proteins of cell lines, cells were directly lysed in SDS-PAGE sample buffer. Western blotting was performed using Clarity Western ECL Substrate (Bio-Rad, CA, USA). The images were obtained with GeneGnome (Syngene, Karnataka, India) and processed with Photoshop CS5.

## Supplementary Information

Supplementary Figures.

## Data Availability

The data sets used during the current study available from the corresponding author on reasonable request.
