# Supplementary material for: The conserved C-terminal residues of FAM83H are required for the recruitment of casein kinase 1 to the keratin cytoskeleton
Source: Sci Rep. 2022 Jul 12;12:11819. doi: 10.1038/s41598-022-16153-y (PMC9276658; doi:10.1038/s41598-022-16153-y)
Supplement: Supplementary file 1 — Supplementary Figures. [file 41598_2022_16153_MOESM1_ESM.pdf]

The conserved C-terminal residues of FAM83H are required for the recruitment of casein kinase 1 to the keratin cytoskeleton

Takahisa Kuga, Naoki Inoue, Kensuke Sometani, Shino Murataka, Minami Saraya, Rina Sugita, Toshinari Mikami, Yasunori Takeda, Masanari Taniguchi, Kentaro Nishida and Nobuyuki Yamagishi

Supplementary figure legends

Fig. S1. Immunofluorescence of human normal keratinocyte PSVK1 cells.

PSVK1 cells were transfected with the plasmids encoding the indicated FAM83H mutants and were analyzed *via* immunofluorescence using antibodies for (a) FAM83H (green), CK1α (red), and keratin 14 (magenta) or (b) FAM83H (green), CK1α (red), and SC-35 (magenta). Nuclei were visualized by DAPI (blue). (b) Magnified images of the nuclei pointed by yellow arrows are shown. White and red scale bars indicate 10 and 2 μm, respectively.

Fig. S2. Keratin proteins in DLD1, HAM1, and PSVK1 cells.

(a) Whole cellular proteins extracted from DLD1, HAM1, and PSVK1 cells were analyzed by Western blotting using antibodies against the indicated proteins. (b) Full-length chemiluminescent images merged with optical images.

Fig. S3. Co-immunoprecipitation of FAM83H mutants possessing and lacking the KL residues.

(a) Lysates for immunoprecipitation were extracted from DLD1 cells transfected with the plasmids encoding the indicated FAM83H mutants. FLAG-tagged FAM83H mutants were precipitated using anti-FLAG antibody-coated Protein G dynabeads. Input lysates and immunoprecipitates were analyzed by Western blotting using antibodies against the indicated proteins. The numbers (kDa) on the right side of panels indicate the electrophoretic positions of the molecular weight marker proteins (DynaMarker Protein MultiColor III, lot No. 012BI06; BioDynamics Laboratory, Tokyo, Japan). The asterisk (*) indicates keratin 8 bands with smaller molecular masses than the expected 53 kDa. (b) Full-length chemiluminescent images merged with optical images.

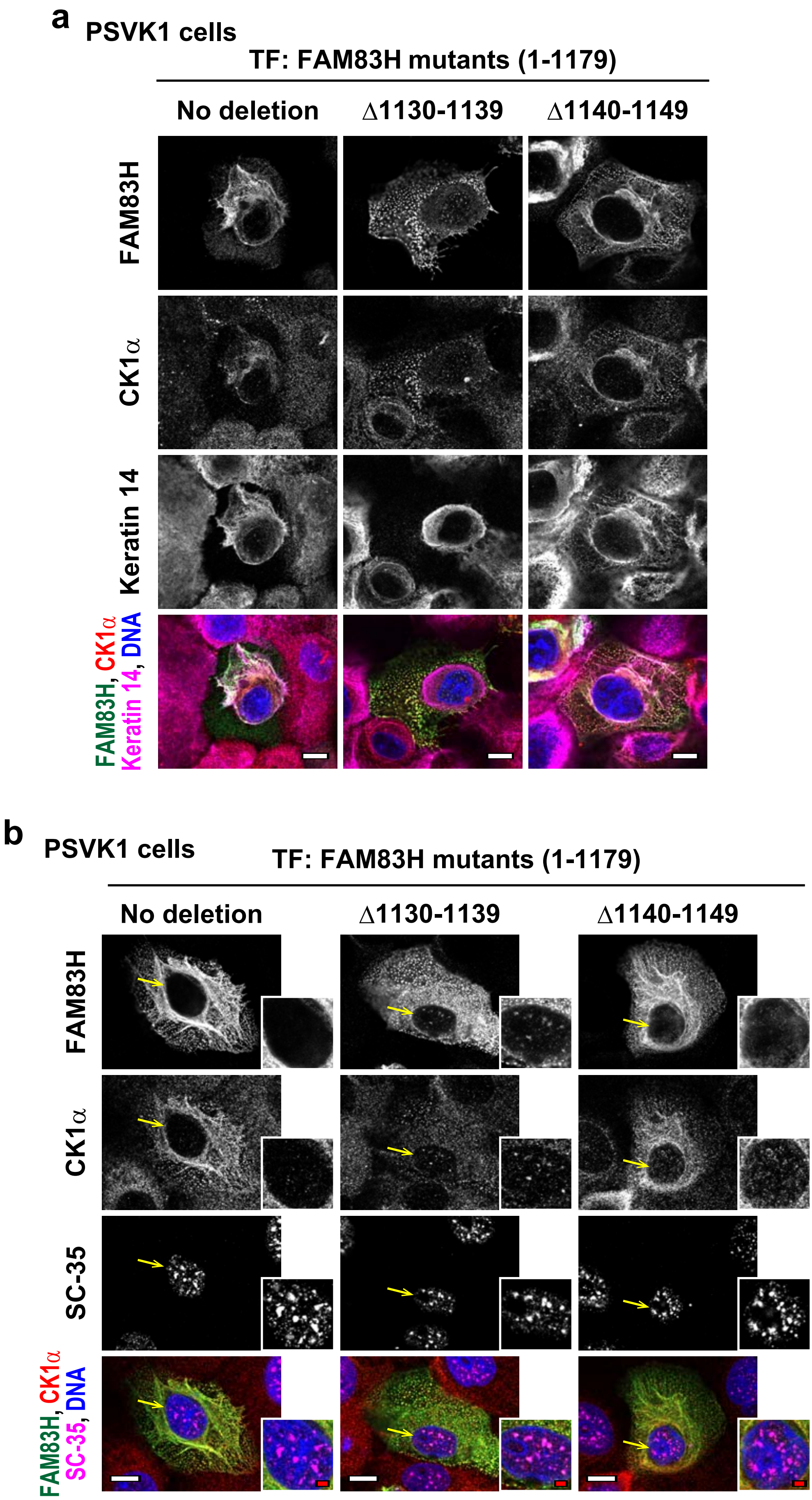
a
PSVK1 cells
TF: FAM83H mutants (1-1179)
No deletion
Δ1130-1139
Δ1140-1149
FAM83H
CK1α
Keratin 14
FAM83H, CK1α, Keratin 14, DNA
b
PSVK1 cells
TF: FAM83H mutants (1-1179)
No deletion
Δ1130-1139
Δ1140-1149
FAM83H
CK1α
SC-35
FAM83H, CK1α, SC-35, DNA

Figure S1

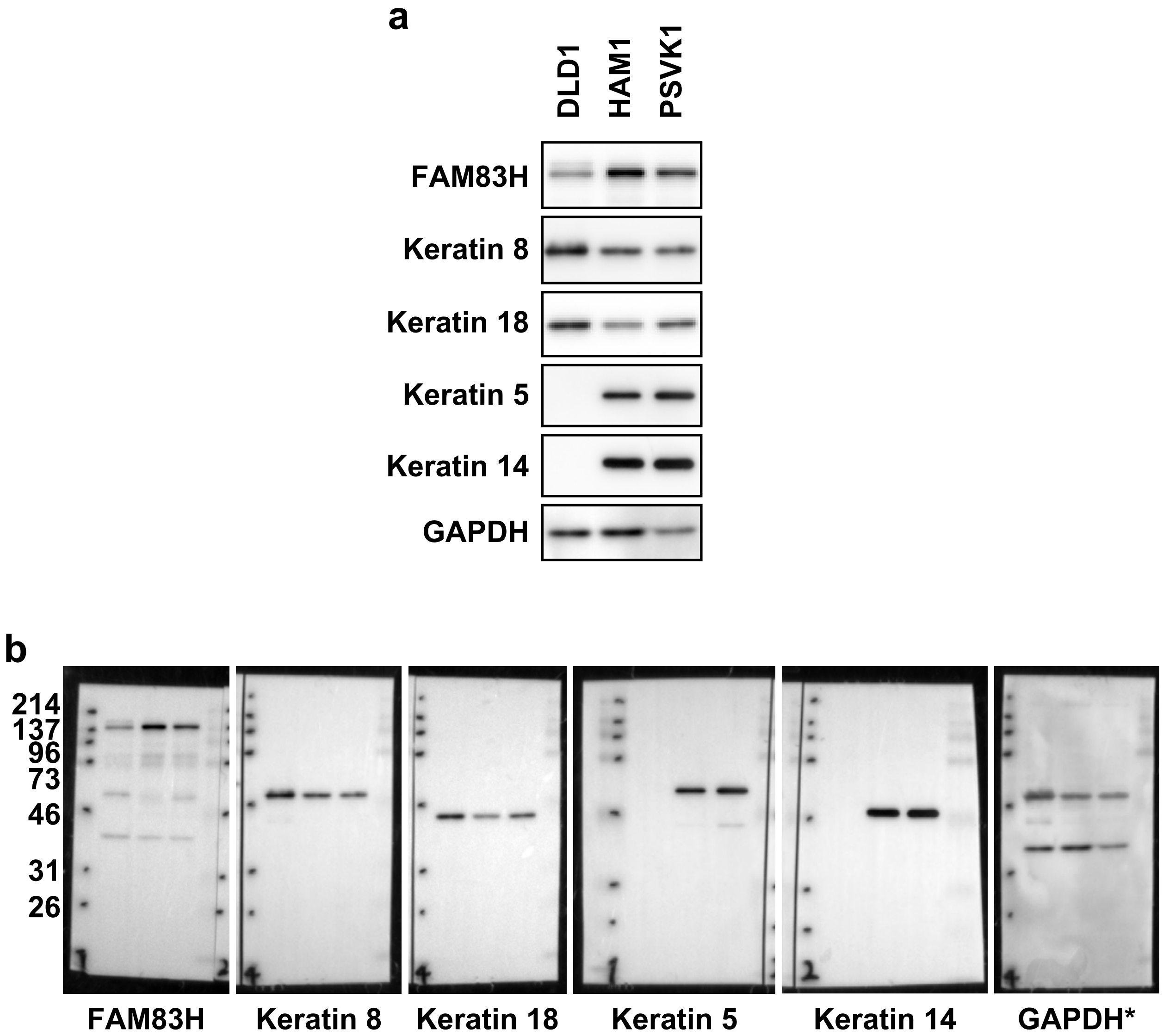

* Reblotting without stripping after blotting for keratin 8

Figure S2

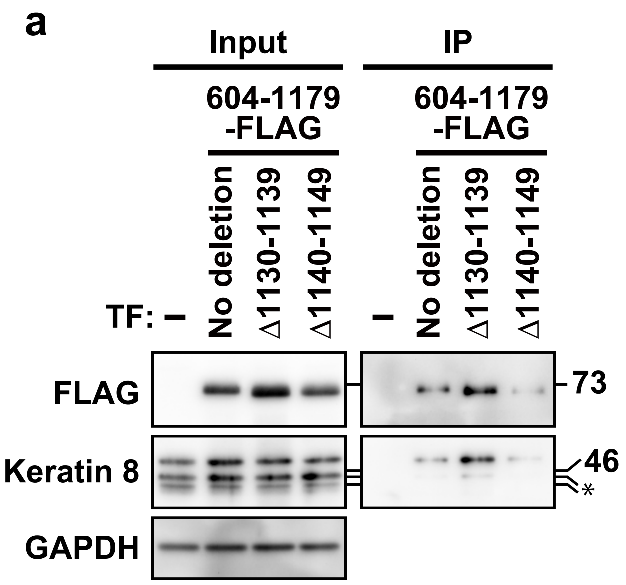

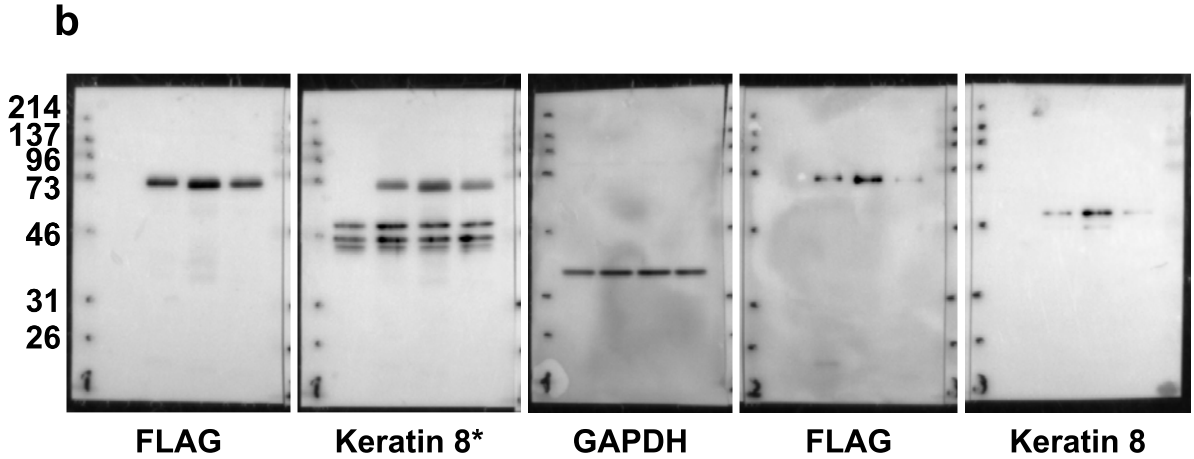

* Reblotting without stripping after blotting for FLAG

Figure S3
